# H5N1 Viruses and Vaccines

**DOI:** 10.1371/journal.ppat.0030040

**Published:** 2007-03-02

**Authors:** Kanta Subbarao, Catherine Luke

**Affiliations:** The Scripps Research Institute, United States of America

The establishment and spread of highly pathogenic avian influenza (HPAI) viruses of the H5N1 subtype in birds and coincident infections in humans since 2003 have raised concerns that we may be facing an influenza pandemic caused by an H5N1 influenza virus. In this brief Opinion piece, we consider the pandemic threat posed by H5N1 viruses and review the published data on the evaluation of H5N1 vaccines in preclinical and clinical studies.

HPAI H5N1 viruses have been isolated from avian species in more than 50 countries. As of 29 January 2007, 270 laboratory-confirmed cases of H5N1 infection in humans had been reported by the World Health Organization, 164 of which were fatal [1], resulting in a case fatality rate of approximately 60%.

In order to cause a pandemic, H5N1 viruses will have to acquire the ability to transmit efficiently from person to person. The H5 hemagglutinin (HA) is found in influenza viruses that typically infect avian species, so efficient person-to-person spread could happen if the H5N1 virus reassorts, or exchanges genes, with circulating human influenza viruses giving rise to a virus with the H5 HA (to which the population is not immune) in a gene constellation that confers the property of transmissibility. Alternatively, efficient person-to-person spread could occur if the H5N1 virus evolves and adapts to more efficient replication and transmissibility in the human population.

Two observations have led to questions about the likelihood of a reassortant H5N1 virus causing a pandemic. First, reassortant viruses have not been isolated despite ongoing H5N1 outbreaks in birds and infections in humans, even with concurrent circulation of human influenza viruses since 2003. Second, laboratory studies have found that reassortant viruses that derived the surface glycoprotein genes from an H5N1 virus and internal protein genes from an H3N2 influenza A virus were not efficiently transmitted and were somewhat less infectious to ferrets (an animal model for human influenza) than the wild-type H5N1 viruses [2]. The concern that an H5N1 virus could adapt to the human host and acquire mutations that confer transmissibility prompts very careful analysis of each cluster of human H5N1 infections that is reported ( [1,3–5]). At present, the data suggest that human-to-human transmission is inefficient and very limited. Nevertheless, from the standpoint of public health preparedness, it is important to move forward in developing approaches for dealing with H5N1 in humans.

Vaccination is the preferred strategy for prevention and control of influenza. The most expeditious way to generate an H5N1 vaccine is to use licensed technology, such as inactivated or live attenuated vaccines. However, several practical and scientific challenges to the development of H5N1 vaccines exist. These include high pathogenicity of wild-type H5N1 influenza viruses, reduced yield of candidate vaccine viruses in embryonated hens' eggs compared to that of human influenza viruses, limited manufacturing capacity, and poor immunogenicity of the H5 HA. Despite these obstacles, several approaches have been used to generate candidate vaccines and a few have advanced to clinical trials (Table 1). Table 1 also includes data published on vaccines that are being developed for veterinary use.

**Table 1:**
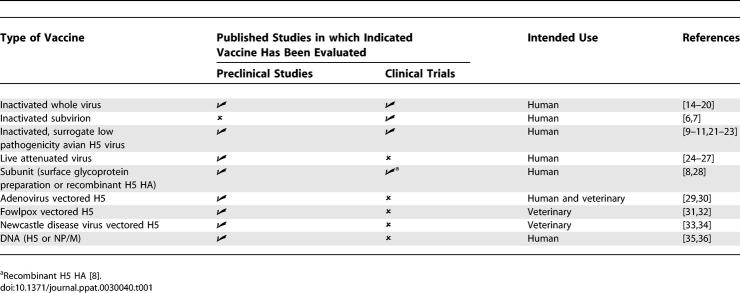
Vaccine Strategies against H5N1 Influenza that Have Been Evaluated in Preclinical and Clinical Studies

Perhaps the most significant scientific challenge for the development and licensure of pandemic vaccines for humans is that assessment of vaccine efficacy for humans will have to be inferred from preclinical studies in experimental animals and immunogenicity studies in humans, as it will not be possible to assess the efficacy of a pandemic vaccine in a clinical trial before a pandemic begins. Table 2 summarizes the preclinical and clinical findings from inactivated H5N1 vaccines evaluated in humans to date. Preclinical studies of influenza vaccines are generally conducted in mice or ferrets. In most cases, the 1997 and 2003 H5N1 vaccine candidates were promising in terms of immunogenicity and efficacy, with complete protection of animals from lethal H5N1 infection, and significant, if not complete, reduction of pulmonary viral replication following challenge. Preclinical data in ferrets have not been published on the 2004 H5N1 vaccines that were evaluated in clinical trials, so data are not available to directly assess how accurately preclinical studies would have predicted the outcome of evaluation of these vaccines in humans.

**Table 2:**
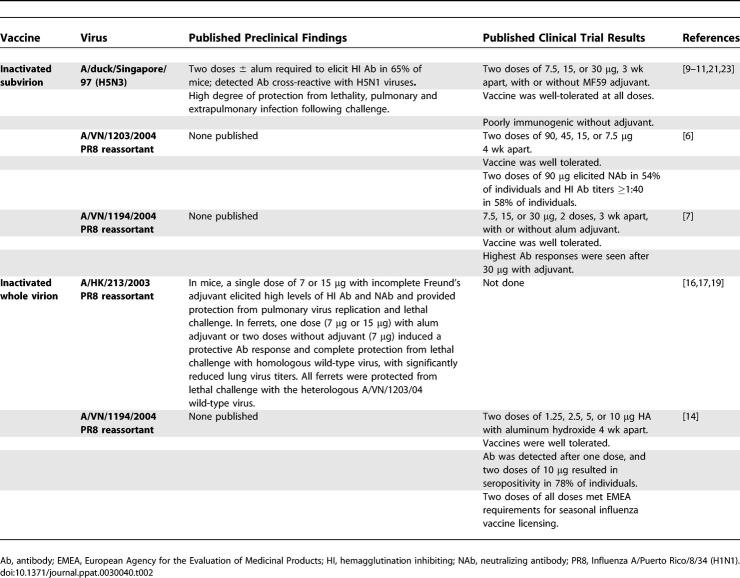
Summary of Preclinical and Clinical Findings for Inactivated H5N1 Virus Vaccines Evaluated in Humans

In clinical trials, inactivated virus vaccines based on H5N1 viruses isolated in 2004 [6,7], a recombinant H5 HA subunit vaccine based on an H5N1 virus isolated in 1997 expressed in a baculovirus vector [8], and an inactivated virus vaccine based on a surrogate low pathogenicity avian H5N3 virus [9–11], were poorly immunogenic when administered to volunteers without adjuvant. Clinical trials of H1N1 influenza vaccines in 1977 established that whole virion vaccines are more immunogenic than split-virion vaccines (in which the virus particles are disrupted by detergent treatment to obtain a preparation enriched for the surface antigens) [12,13]; however, the former are also more reactogenic than the latter. Consistent with this observation, in recent trials in humans of an alum-adjuvanted inactivated H5N1 virus vaccine, much lower doses of a whole virion vaccine elicited higher levels of antibody compared to a split-virion vaccine [7,14]. Despite the fact that the difference in immunogenicity of whole virion and split-virion vaccines was well established, preclinical studies of inactivated H5 virus vaccines in mice and ferrets have generally been performed using whole virion preparations with adjuvant, while the vaccine preparation evaluated in clinical trials is a purified split-virion vaccine. It is important to note that preclinical data will not be predictive of clinical trial results if the vaccine formulations that are tested in preclinical studies are different from those evaluated in clinical trials.

Clinical trials have demonstrated that the immunogenicity of H5 vaccines can be enhanced by an increased dose of the HA, the use of adjuvants, use of multiple doses, or use of a whole virion vaccine. More studies are needed to directly compare findings from preclinical and clinical evaluation of pandemic influenza vaccines to establish whether animal models can be used to guide decisions on which vaccine candidates to take forward for evaluation in humans. Although there is no evidence that H5N1 viruses have yet acquired pandemic potential, the consequences of such an event are serious enough that preparation for a possible pandemic is essential. 

## Abbreviation

HA: hemagglutinin
